# Assessment of barriers to pancreatic cancer surveillance in high‐risk individuals

**DOI:** 10.1002/jgc4.70117

**Published:** 2025-10-09

**Authors:** Grace G. Snyder, Daniel Clay, Sara Karley, Samantha Pipito, Rebecca Mueller, Angela Bradbury, Kara Maxwell, Katherine L. Nathanson, Mersedeh Rohanizadegan, Payal Shah, Susan M. Domchek, Jessica M. Long, Bryson W. Katona

**Affiliations:** ^1^ Genetic Counseling Program University of Pennsylvania Philadelphia Pennsylvania USA; ^2^ Division of Gastroenterology and Hepatology University of Pennsylvania Philadelphia Pennsylvania USA; ^3^ Division of Hematology and Oncology University of Pennsylvania Philadelphia Pennsylvania USA; ^4^ Division of Translational Medicine and Human Genetics University of Pennsylvania Philadelphia Pennsylvania USA

**Keywords:** cancer genetic counseling, cancer risk management, content analysis, health decisions, pancreatic cancer surveillance, patient‐centered care, service delivery barriers

## Abstract

Individuals with increased familial or genetic risk of pancreatic cancer (PC) may be recommended to undergo regular PC surveillance. Genetic counselors are often involved in discussions about PC surveillance for high‐risk individuals (HRIs); however, barriers to HRIs' participation in PC surveillance are not well characterized. This study aimed to identify reasons that HRIs cease, defer, or do not commence recommended PC surveillance through telephone interviews. Participants either had prior annual PC surveillance with no surveillance completion in ≥2 years, had a ≥2‐year period without surveillance completion, or had not completed an initial surveillance imaging study 3 months after it was ordered. Fifty telephone interviews were analyzed using directed content analysis. Twenty interviewees had familial PC (34.5%) and 38 (65.5%) had a pathogenic variant associated with increased PC risk, with *BRCA2* being the most common (*N* = 15, 25.9%). Interviewees were 74.1% women and 93.1% White with a median age of 63.0 years. Logistical barriers (*N* = 11, 34.4%), different healthcare professional recommendations (*N* = 9, 28.1%), other health issues (*N* = 8, 25.0%), and difficulty recalling surveillance recommendations (*N* = 8, 25.0%) were the top reasons for ceasing or deferring PC surveillance. Difficulty recalling surveillance recommendations (*N* = 5, 27.8%), cost (*N* = 4, 22.2%), and invasiveness of procedures (*N* = 4, 22.2%) were the top reasons for not commencing PC surveillance. Other reasons included the COVID‐19 pandemic, moving from the service delivery area, cost, concerns about imaging studies, nonmedical life events, and fear. Several barriers identified in this study were consistent with barriers faced in screening for other more common cancers. These results demonstrate the need for targeted strategies to reduce PC surveillance barriers for HRIs. Furthermore, given that HRIs face multiple barriers to PC surveillance, it is important that cancer genetics professionals familiarize themselves with these barriers to reduce their impact and to facilitate recommended PC surveillance among HRIs.

**What is known about this topic**

Individuals with familial or genetic risk for pancreatic cancer may be recommended to undergo regular pancreatic cancer surveillance. Pancreatic cancer surveillance can improve pancreatic cancer outcomes through early detection; however, some high‐risk individuals do not complete recommended surveillance.

**What this paper adds to the topic**

The barriers to high‐risk individuals participating in PC surveillance are not well characterized. This study identified commonly reported reasons that high‐risk individuals cease, defer, or do not commence pancreatic cancer surveillance, thus increasing awareness of these barriers among cancer genetics professionals and facilitating action by healthcare professionals to help reduce barriers for HRIs.

## 1 INTRODUCTION/BACKGROUND

Pancreatic cancer (PC) is the third‐most common cause of cancer death in women and the fourth‐most common cause of cancer death in men in the United States; it is predicted to become the second‐most common cause of cancer death by 2040 (Rahib et al., 2021; Siegel, 2025). PC has a poor prognosis as many cases are detected at an advanced stage of disease, and with an overall 5‐year survival rate of 13%, PC has one of the lowest survival rates among cancers in the United States (Siegel, 2025). Up to 15% of PC is due to hereditary risk, including 3%–5% that is attributable to known genetic cancer predisposition syndromes, and 5%–10% that is diagnosed in individuals who have familial risk without a known genetic cause; persons belonging to either category are considered high‐risk individuals (HRIs) (Owens et al., 2019). Germline pathogenic variants (PVs) in *BRCA1*, *BRCA2*, *ATM*, and most Lynch syndrome genes (*MLH1*, *MSH2*, *MSH6*, and *EPCAM*) are associated with an increased risk of PC. PVs in *CDKN2A*, *PRSS1*, *STK11*, *TP53*, and *PALB2* are less common but are also associated with increased PC risk (Seppälä et al., 2023; Wood et al., 2019). Familial PC is defined as a family with two directly related relatives with PC in the absence of a known genetic cause (Goggins et al., 2020).

Early detection of PC has been associated with improved outcomes in some studies, but universal screening for PC is not recommended largely due to the low incidence of PC in the general population; PC accounted for only 3.3% of all new cancer cases in 2024 (Blackford et al., 2020; Klatte, 2023; Owens et al., 2019; Siegel, 2024). Additionally, debate about the best screening modality, limitations of the imaging studies used to screen for PC, and the potential harms associated with screening together outweigh the potential benefits in asymptomatic, average‐risk populations. Thus, the U.S. Preventive Services Task Force (USPSTF) recommends against PC screening in asymptomatic, average‐risk adults (Owens et al., 2019). However, recent guidelines recommend consideration of annual PC surveillance in appropriately aged HRIs by abdominal MRI with magnetic resonance cholangiopancreatography (MRCP) or endoscopic ultrasound (EUS) (Goggins et al., 2020; NCCN, 2025; Sawhney et al., 2022). For those with familial PC in the absence of a known hereditary syndrome, appropriate age for surveillance onset, while debated in the literature, has been suggested as 45–55 years old or 10 years younger than the earliest familial PC. Individuals with PVs in certain “lower penetrance” genes such as *BRCA1*, *BRCA2*, or *ATM* may similarly consider starting surveillance at 50 years old or 10 years younger than the earliest onset exocrine PC in a relative, although there is variability in published guidelines about when to begin PC surveillance for individuals with a PV in one of these genes, or if surveillance should be started at all (NCCN, 2025). For HRIs with highly penetrant, autosomal dominant cancer predisposition syndromes that are associated with earlier‐onset PC, such as Peutz‐Jeghers syndrome (*STK11*) or familial atypical multiple mole melanoma syndrome (FAMMM syndrome, *CDKN2A*), surveillance may begin younger at 30–35 or 40 years old, respectively (Goggins et al., 2020; NCCN, 2025; Sawhney et al., 2022; Syngal et al., 2015).

The benefits of PC surveillance, including downstaging PC at diagnosis and improved overall survival, have been demonstrated in some studies (Dbouk et al., 2022; Klatte, 2023). In one study of 1731 HRIs, among 26 surveillance‐detected PCs, 58% were stage I with a 5‐year survival rate of 73.3% (Dbouk et al., 2022). For comparison, only 10% of PCs diagnosed in the U.S. for those not undergoing surveillance are stage I (Blackford et al., 2024). Still, the benefits of annual pancreatic surveillance must be weighed against the risks of concomitant invasive diagnostic procedures, the potential for surgical intervention for benign lesions, financial costs, and time burdens.

Previous studies have evaluated factors affecting compliance with cancer surveillance among individuals at high risk for other cancers (e.g., breast, colon); however, poor prognosis, lack of reliable surveillance, and the relatively high morbidity of potentially curative surgery present unique challenges for PC surveillance compared with other cancer surveillance programs (Fendrich et al., 2014). Despite existing PC surveillance recommendations, surveillance of HRIs has not been widely adopted (Zogopoulos et al., 2024). Furthermore, there is limited understanding about the factors impacting HRIs' engagement with recommended PC surveillance. Franke et al. (2018) found that many HRIs cease PC surveillance, and the International Cancer of the Pancreas Screening Consortium suggests that further study is required on factors influencing engagement with PC surveillance (Goggins et al., 2020).

Cancer genetic counselors are often one of the first healthcare professionals to address the topic of PC surveillance with HRIs and often receive questions about this topic, even after cancer risk assessment and genetic testing are completed. Therefore, understanding the factors that influence engagement with PC surveillance is critical for adopting equitable policies in healthcare management that support patient engagement and access to recommended surveillance following identification of increased hereditary risk to develop PC. To address this area of need, our study herein explores factors influencing PC surveillance engagement in a population of HRIs by identifying reasons for cessation, deferral, or noncommencement of PC surveillance. In addition, we aimed to identify barriers to care that may be addressed during shared decision‐making between HRIs and healthcare professionals to potentially facilitate patient re‐engagement in PC surveillance.

## 2 METHODS

This study used qualitative, semi‐structured interviews to investigate barriers to PC surveillance. A positivist framework and directed content analysis were used to deductively and inductively analyze the interview data.

### 2.1 Study population

**FIGURE 1:**
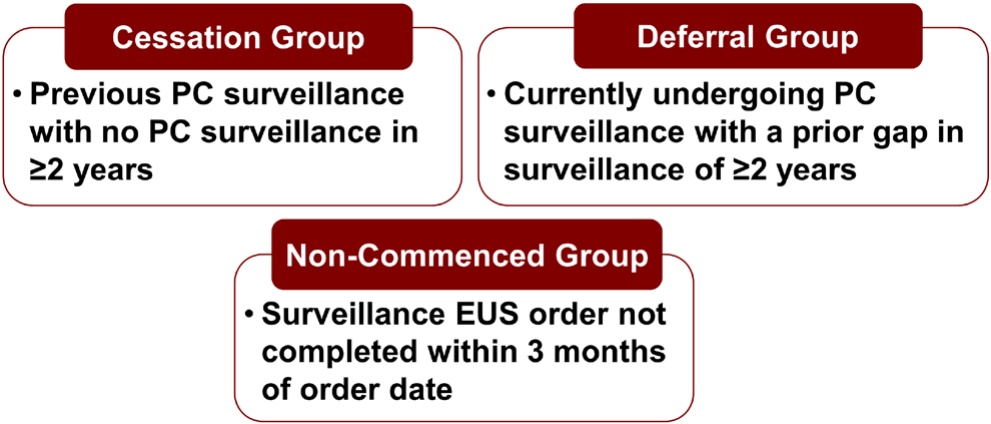
Eligibility criteria for study participants. Participants in the cessation group had previously undergone PC surveillance but had no PC surveillance imaging studies in two or more years. Participants in the deferral group were currently undergoing PC surveillance but had a prior gap of two or more years in which they did not complete a PC surveillance imaging study. Participants in the noncommenced group had not yet started PC surveillance but had active EUS orders that were neither scheduled nor completed within 3 months of the order date.

Two groups of potential study participants were ascertained from individuals enrolled at Penn Medicine in at least one of three ongoing PC surveillance studies, comprising 563 HRIs at the time of ascertainment. These three studies included an institutional PC surveillance study [NCT02478892], CAPS5 [NCT02000089], and PRECEDE [NCT04970056]. Individuals were eligible for participation in the current study if they met one of the following criteria: “cessation” group participants previously had a PC surveillance imaging study and were eligible for continued annual surveillance, but had not completed a PC surveillance imaging study within the past 2 years; “deferral” group participants had a period of at least 2 years (between January 1, 2017 and July 1, 2024) wherein recommended surveillance imaging was not completed, even if the individual was currently up to date with PC surveillance (Figure 1).

A third group of potential participants (“noncommenced” group) was ascertained via an electronic medical record (EMR) data request that identified individuals with active EUS orders who also had an active Genomic Indicator designating a confirmed PV in *BRCA1*, *BRCA2*, *PALB2*, *ATM*, *STK11*, *CDKN2A*, *MLH1*, *MSH2*, *MSH6*, or *EPCAM*. At the study site, appropriately aged individuals with a PV identified in *BRCA1*, *BRCA2*, *PALB2*, *ATM*, *STK11*, or *CDKN2A* may be offered PC surveillance, typically with EUS, regardless of whether the individual has a known family history of PC. Appropriately aged individuals at the study site with a PV in *MLH1*, *MSH2*, *MSH6*, or *EPCAM* may be offered PC surveillance if they have a first‐ or second‐degree relative with PC; as there is not evidence demonstrating that PVs in *PMS2* confer an increased risk of PC, PC surveillance recommendations for *PMS2* carriers are based on other genetic predisposition and/or family history alone, independent of the *PMS2* PV (NCCN, 2025). These individuals had not yet undergone any PC surveillance following recommendation by their physician to initiate PC surveillance. Individual participant charts were manually reviewed by the research team to validate study eligibility, which required an active EUS order that remained incomplete and unscheduled at least 3 months from the date the order was placed. Participants were excluded if less than 3 months had elapsed since the EUS was ordered.

Qualifying PC surveillance imaging studies for participants in the cessation and deferral groups included EUS or abdominal MRI with IV contrast. For the noncommenced group, EUS only was applied as a filter during the initial data request because EUS is typically performed as the baseline PC surveillance imaging study at the study site and is ordered for a narrower range of indications. All imaging studies and orders were verified via manual review of each potential participant's EMR at the study site.

### 2.2 Recruitment

Eligible participants were called by the first author, a female genetic counseling student with no prior relationship to any participants, at the telephone number listed in their EMR profile. The first author described the study to participants and obtained their verbal informed consent to participate in a brief interview. Individuals who did not answer the initial telephone call were sent a follow‐up message with information about the study via the EMR patient portal. Individuals who neither answered the initial phone call nor responded to the EMR message were contacted again via phone at least 4 weeks after the initial attempt. This study was approved by the University of Pennsylvania Institutional Review Board on 16 April 2024 (protocol # 855599).

### 2.3 Data collection

Semi‐structured interviews were conducted with participants who had not completed an interval (cessation or deferral groups) or baseline (noncommenced group) PC surveillance imaging study at the study site. The interview guide was developed by experts in cancer genetics and PC surveillance with the intention of investigating patient actions and decisions with respect to PC surveillance (Data S1). Specific questions were developed through iterative discussion with the study team.

Following verbal informed consent, participants were first asked whether any surveillance imaging studies had occurred at a facility outside of the study site to validate the data collected from the EMR. Participants then answered open‐ended questions about medical, personal, and financial factors impacting PC surveillance decisions. The interview guide included probes in each domain, and each participant was asked additional exploratory questions at the discretion of the interviewer.

Interviews lasted from 2 to 30 minutes, with the interview time starting after informed consent was obtained and ending when the final question of the interview guide had been answered. Interviews were transcribed in real time using Microsoft Word's built‐in Office Dictation software for participants consenting to software use. Hand‐typed notes were taken for participants who did not consent to transcription software use. Immediately following each interview, the dictated transcripts were cleaned for accuracy and readability with added annotations regarding participant tone and affect. For participants declining use of transcription software, hand‐typed notes were written during and immediately following the interview. Each transcript was assigned a unique study ID number to maintain confidentiality.

### 2.4 Data analysis

Interviews were analyzed using directed content analysis (Hsieh & Shannon, 2005; Lynch et al., 2024). Deductive codes were developed based upon clinical observations of the study team and prior literature that identified factors associated with discontinuation of PC surveillance and colon cancer surveillance (Franke et al., 2018; Nagelhout et al., 2017). We created additional inductive codes for interview responses that could not be categorized with the initial coding scheme, as described by Hsieh and Shannon (2005). Cleaned interview transcripts were reviewed by the first author to apply deductive codes and to develop an initial set of inductive codes, which were then reviewed and modified by the study team. Transcripts were subsequently independently re‐coded in Dedoose (2025) by the first author using both the deductive and inductive codes. A second team member then coded the transcripts, and coding differences were reconciled by discussion. Summary statistics, including medians and percentages, were used to describe the demographic characteristics of the participants. Summary statistics were also used to describe reasons for PC surveillance cessation, deferral, or noncommencement.

## 3 RESULTS

### 3.1 Study cohort

Of the 96 potential participants identified, 41 (42.7%) were from the cessation group, 16 (16.7%) were from the deferral group, and 39 (40.6%) were from the noncommenced group (Table 1). Twenty‐eight potential participants (29.2%) had familial PC and 68 (70.8%) had a PV in a PC risk gene, including *BRCA2* (*N* = 32, 33.3%), *BRCA1* (*N* = 12, 12.5%), *ATM* (*N* = 8, 8.3%), a Lynch syndrome gene (*N* = 6, 6.3%), *CDKN2A* (*N* = 5, 5.2%), *PALB2* (*N* = 4, 4.2%), or *STK11* (N = 1, 1.0%). Of the total 68 potential participants who had a PV in a PC risk gene, 30 had a positive family history and 38 had a negative or unknown family history of PC (Table S1). Fifty‐eight of the 96 eligible participants consented and completed an interview for a response rate of 60.4%. The cohort of 58 participants that completed interviews was majority female (74.1%) and predominantly White (93.1%) with a median age of 63.0 years (IQR = 17 years). The 38 participants that did not complete an interview were 78.9% female and 84.2% White with a median age of 67.5 years (IQR = 15.5 years). Twenty‐six of the 58 interviewed participants (44.8%) were in the cessation group, 11 (19.0%) were in the deferral group, and 21 (36.2%) were in the noncommenced group.

**TABLE 1:** Study population demographics. In total, there were 96 potential participants with 58 of these individuals participating in an interview. Fifty‐seven potential participants were identified during the recruitment process from a pool of 563 individuals enrolled in an ongoing PC surveillance study at Penn Medicine at the time of participant ascertainment. Thirty‐nine participants were identified from an EMR data request.

	Total	Participated in interview	Did not participate
	*N* = 96	*N* = 58	*N* = 38
Age, median (IQR)	65.0 (16)	63.0 (17)	67.5 (15.5)
Sex, *N* (%)			
Female	73 (76.0%)	43 (74.1%)	30 (78.9%)
Male	23 (24.0%)	15 (25.9%)	8 (21.1%)
Self‐reported race			
White	86 (90.0%)	54 (93.1%)	32 (84.2%)
Black or African American	6 (6.3%)	2 (3.4%)	4 (10.5%)
Other or unknown	3 (3.1%)	2 (3.4%)	1 (2.6%)
Asian	1 (1.0%)	0 (0%)	1 (2.6%)
PV in PC risk gene	68 (70.8%)	38 (65.5%)	30 (80.0%)
*BRCA2*	32 (33.3%)	15 (25.9%)	17 (44.7%)
*BRCA1*	12 (12.5%)	8 (13.8%)	4 (10.5%)
*ATM*	8 (8.3%)	7 (12.1%)	1 (2.6%)
Lynch syndrome (*MLH1*, *MSH2/EPCAM*, or *MSH6*)	6 (6.3%)	3 (5.2%)	3 (7.9%)
*CDKN2A*	5 (5.2%)	1 (1.7%)	4 (10.5%)
*PALB2*	4 (4.2%)	3 (5.2%)	1 (2.6%)
*STK11*	1 (1.0%)	1 (1.7%)	0 (0%)
Familial PC	28 (29.2%)	20 (34.5%)	8 (21.1%)
Group			
1. Cessation (no surveillance in ≥2 years)	41 (42.7%)	26 (44.8%)	15 (39.5%)
2. Deferral (gap in surveillance of ≥2 years)	16 (16.7%)	11 (19.0%)	5 (13.2%)
3. Noncommenced (surveillance EUS not scheduled nor completed)	39 (40.6%)	21 (36.2%)	18 (47.4%)

**FIGURE 2:**
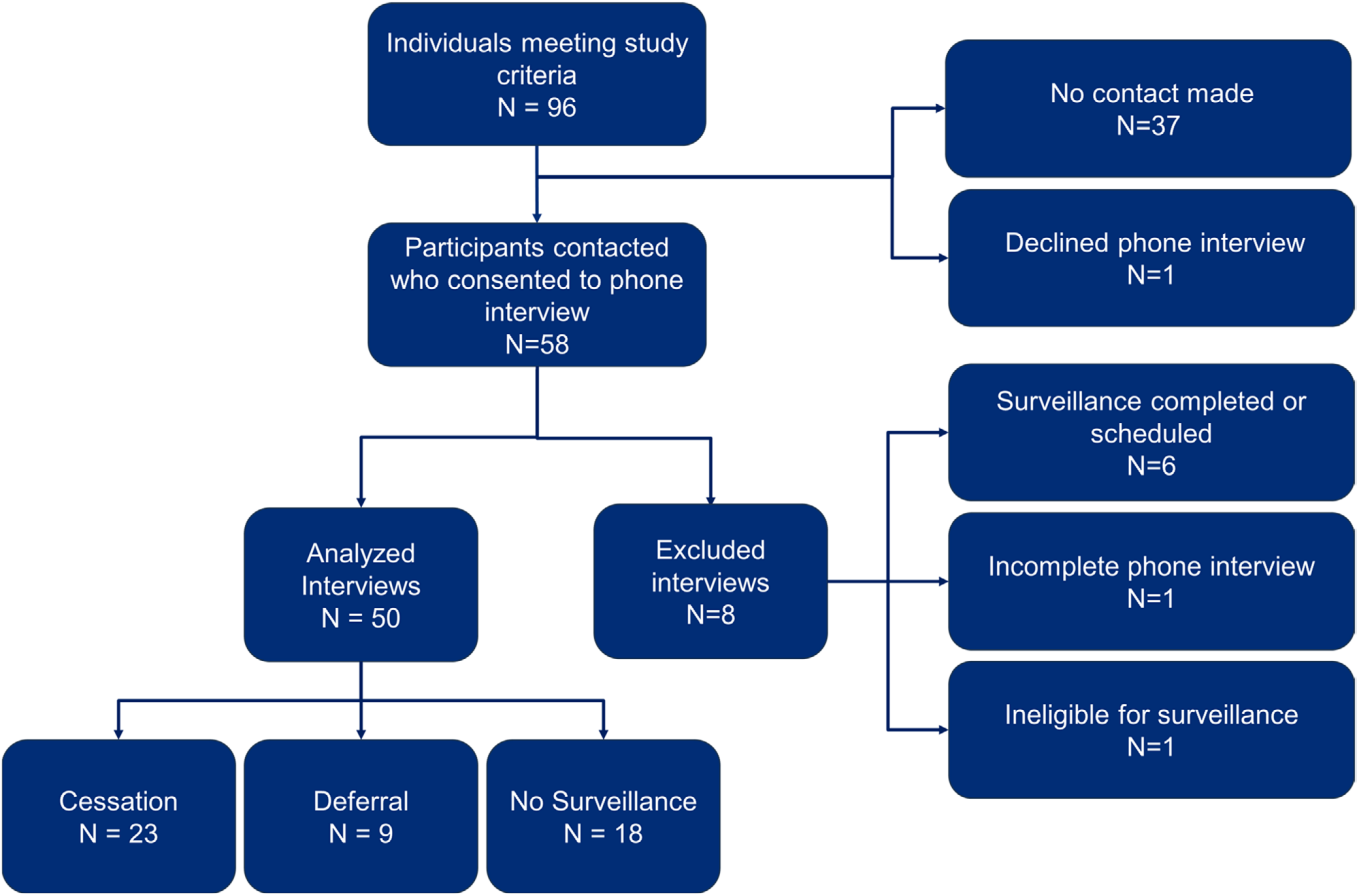
Participant contact. Fifty‐eight participants were reached and consented for interviews; 50 interviews were included in the analysis. Eight participants were found to not meet inclusion criteria at the time of interview, and their interview responses were excluded from analysis. Of the eight excluded interviews, three were from the cessation group, two were from the deferral group, and three were from the noncommencement group.

Six of the 58 interviewed participants (10.3%) reported a completed or scheduled surveillance imaging study at a location other than the study site (Figure 2). One participant (1.7%) had performed a baseline EUS at the time of positive genetic testing for a PV in a gene associated with an increased risk of PC but did not meet current eligibility criteria for yearly surveillance. One other participant (1.7%) was not able to fully complete their interview. The interview responses of these eight participants were excluded from further analysis.

### 3.2 Reported reasons for PC surveillance cessation or deferral

**FIGURE 3:**
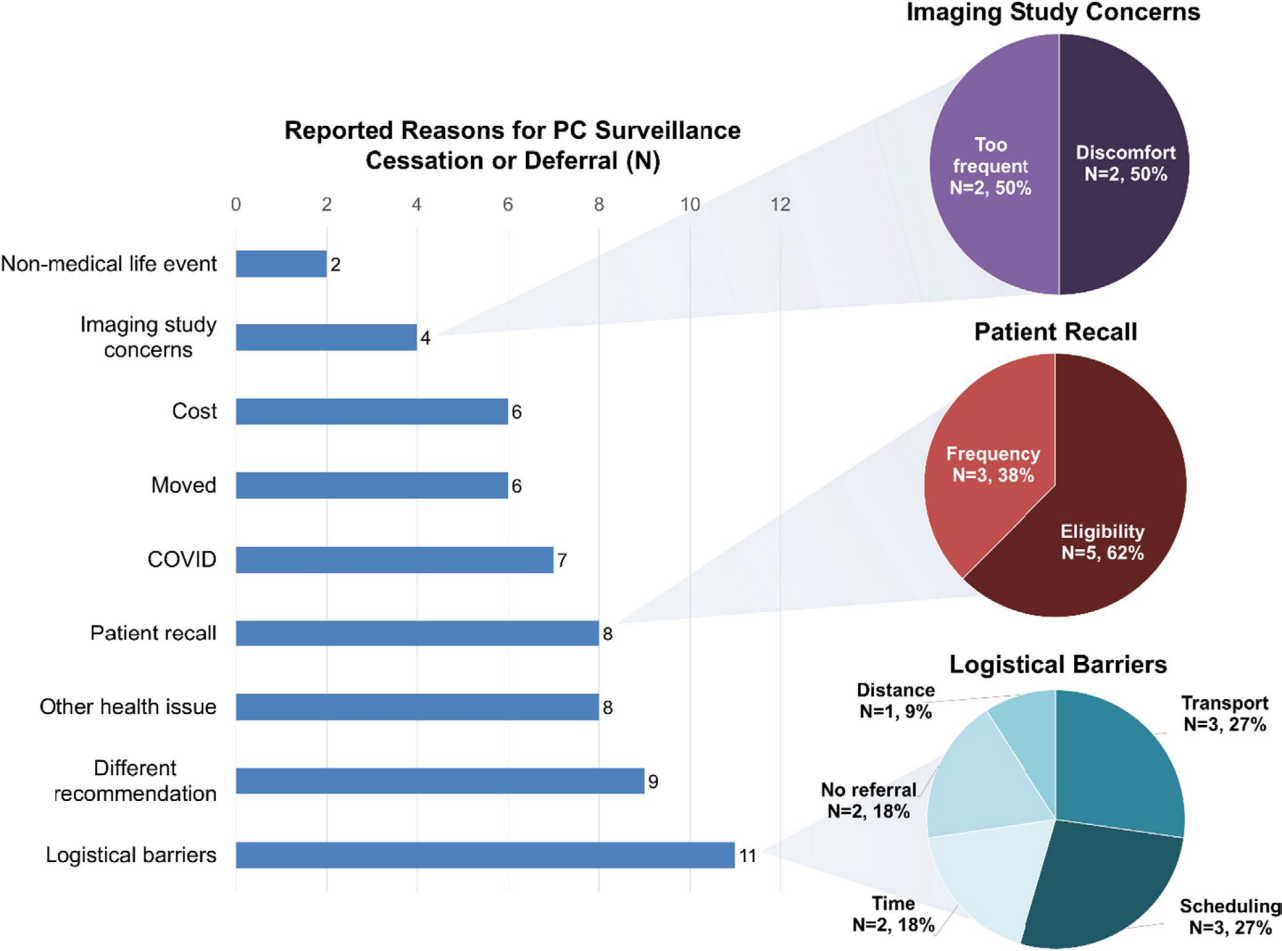
Reasons cited by participants for PC surveillance cessation or deferral. All participants (*N* = 32) reported ≥1 reason(s). Types of imaging study concerns (top right), patient recall discrepancies (middle right), and reported logistical barriers (bottom right) are also shown.

Reasons for stopping or deferring PC surveillance are shown in Figure 3. Logistical barriers (relating to transport, time, scheduling, referral availability, or distance) were reported by 11 participants. For example, one participant explained, “I did [surveillance] for about 10 years and then my husband passed away and I stopped… He drove me and once he passed, I wasn't going to the city to get them.”—Study ID 44, cessation group, familial PC. The breakdown of all reported logistical barriers for the cessation and deferral groups is displayed in Figure 3.

Nine participants (28.1%) reported receiving a different recommendation from another healthcare professional about the frequency of PC surveillance, in contrast to the recommendation documented at the time of the participant's enrollment in a PC surveillance study. Different recommendations between healthcare professionals included expanding surveillance intervals from annually to every 2 years, only performing EUS in conjunction with a colonoscopy, or ceasing surveillance altogether. One participant shared, “That's where I feel like the ball is really dropped, you know? I have one gastroenterologist tell me, ‘Just because your dad had [pancreatic cancer] do not panic, that doesn't mean you [are] going to have it.’ When I mentioned it to other longtime doctors I've had, even my gynecologist, people I've had since my 20s, they gasp, and I'm like ‘Oh my God, you're making me panic!’ So, I feel like I haven't gotten consistent advice.”*—*Study ID 55, deferral group, familial PC. Another participant recommended to undergo annual PC surveillance via MRI or EUS described her experience discussing PC surveillance at her new gastroenterologist's office, explaining “I mentioned [PC surveillance] to the gastro guy that I go to now, but he didn't seem concerned about it. Let's put it that way.”—Study ID 22, cessation group, familial PC.

Eight participants (25.0%) reported prioritization of competing health concerns over continued PC surveillance. One participant recollected, “I believe I'm supposed to go every year, but what happened is I had lung cancer… I've had a lot of surgeries lately, but normally I try to get it.”—Study ID 31, cessation group, PV in a PC risk gene.

Eight participants (25.0%) either did not know they were eligible for continued PC surveillance or could not recall a specific recommendation from their physician about frequency and/or modality of recommended surveillance, even if they knew they were eligible. For example, one participant exclaimed, “It's funny that you called just now because I just asked [my doctor] if I'm supposed to go, like when do I go again for colonoscopy or the EUS? I had no idea, so great that you're calling!”—Study ID 20, deferral group, PV in a PC risk gene.

Other barriers included the COVID‐19 pandemic (*N* = 7, 21.9%), moving out of the service delivery area (*N* = 6, 18.8%), cost (*N* = 6, 18.8%), imaging study‐related concerns such as EUS invasiveness, difficulty tolerating the imaging study, or concerns about its frequency (*N* = 4, 12.5%), and nonmedical life events (*N* = 2, 6.3%). One participant summarized, “Life is busy, I work full time, I have kids. My mom passed away two years ago and I was caring for her, that sort of stuff, time slips by.”—Study ID 12, deferral group, PV in a PC risk gene.

All participants reported at least one reason for deferring or ceasing PC surveillance. Of the analyzed cessation group interviews (*N* = 23), three participants (13.0%) expressed interest in re‐engaging in surveillance and were connected upon request to the appropriate clinical team.

### 3.3 Reported reasons for not yet commencing PC surveillance

**FIGURE 4:**
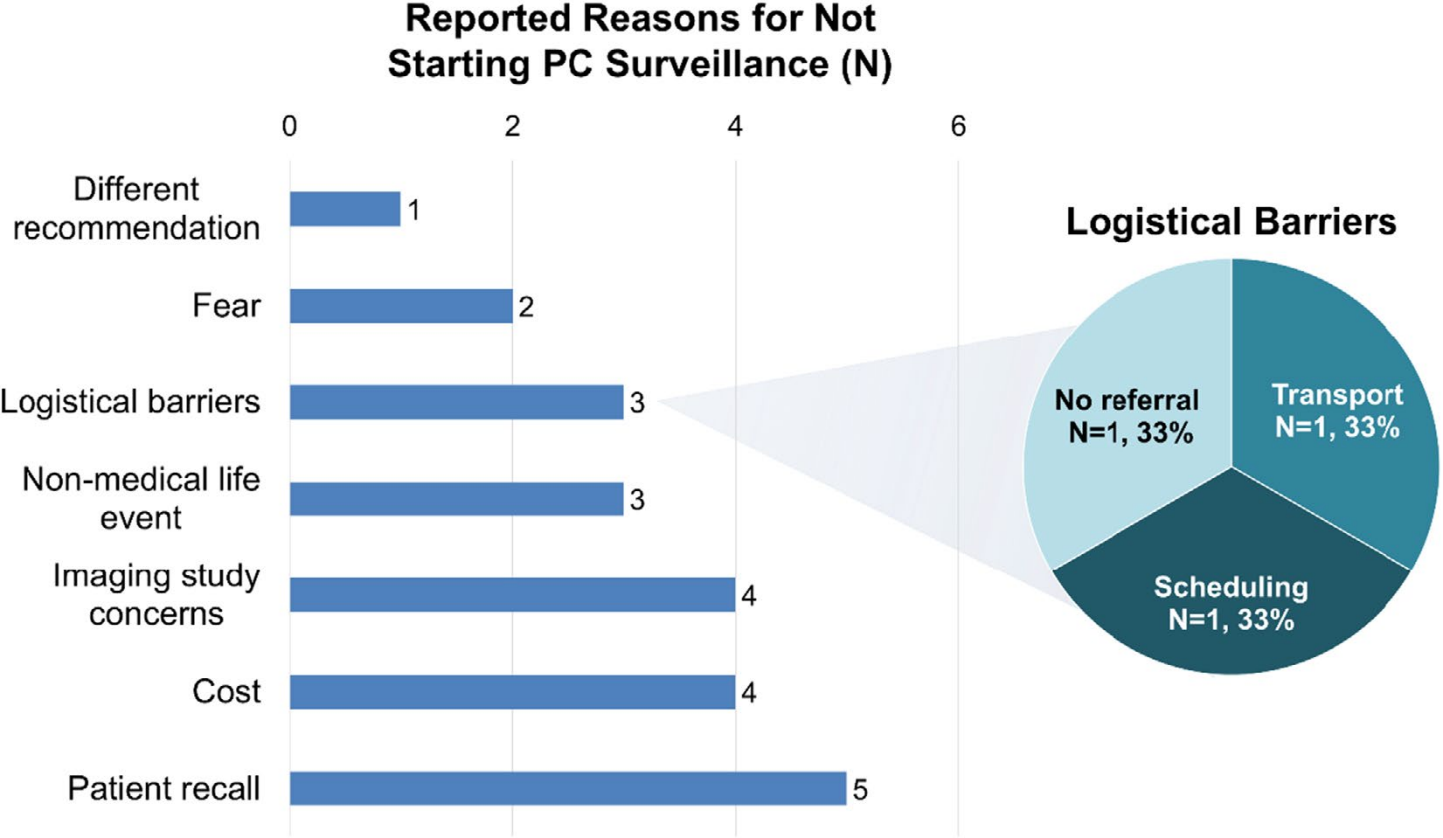
Reasons participants cited for not commencing PC surveillance. All participants (*N* = 18) reported ≥1 reason(s). Of the three participants reporting logistical barriers, one each reported being too distant from the study site, difficulty scheduling, or having no transport.

Reasons for not commencing PC surveillance are shown in Figure 4. All reasons reported by participants in the noncommenced group were the same as those reported in the cessation and deferral groups, except for two participants who reported generalized feelings of fear as the reason for not starting PC surveillance. As one participant explained, “It was just me thinking I didn't want to poke the bear.”—Study ID 59, noncommenced group, PV in a PC risk gene. No participants in the noncommenced group expressed interest in moving forward to schedule surveillance during the phone interview.

Of the 18 interviews analyzed from participants who had not yet started PC surveillance, five participants (27.8%) did not recall that they were recommended to begin PC surveillance. Four participants (22.2%) reported cost as the primary reason for not beginning PC surveillance. Another four participants (22.2%) reported the imaging study‐related concern that EUS was too invasive. Other reasons for not starting surveillance included nonmedical life events (*N* = 3, 16.7%), logistical barriers (*N* = 3, 16.7%), fear (*N* = 2, 11.1%), or different recommendations from a healthcare professional (*N* = 1, 5.6%).

## 4 DISCUSSION

This study assessed barriers to PC surveillance, specifically, the reasons HRIs deferred, ceased, or did not commence PC surveillance. Our results demonstrated that for HRIs in our study who ceased, deferred, or did not commence surveillance, the reasons for not participating included logistical barriers, differing healthcare professional recommendations, difficulty recalling surveillance recommendations, and other health concerns, among other reasons. Broadly speaking, we identified barriers that can be considered directly actionable, potentially actionable, or have limited/no actionability by healthcare professionals. Our findings, combined with findings from the literature, suggest that the most common barriers to PC surveillance, including certain logistical barriers, patient recall, and to some extent, differences between imaging study recommendations, are directly actionable and can be addressed with interventions from healthcare institutions. Imaging study concerns or patient fear are potentially actionable and may be addressed by appropriate healthcare professionals, including mental health professionals. Other barriers, such as cost, medical or nonmedical life events, moving, and COVID‐19, are less directly actionable for healthcare teams working with HRIs. However, the burden of these barriers may be lessened by the development of noninvasive PC surveillance tools, such as blood‐based tests, that would be more accessible compared with current surveillance modalities.

Some logistical barriers have straightforward, actionable interventions, while others are more complex though still actionable. Difficulty scheduling may be addressed by allocating additional resources to scheduling staff or to the development of online scheduling. One study of individuals with Lynch syndrome found that reminders to schedule imaging studies and ease of scheduling appointments were the two most cited facilitators to continued surveillance (Mooney et al., 2023). Additionally, transportation limitations are repeatedly cited as barriers to healthcare access, especially for older adults facing social isolation or loss of loved ones, and it is important to consider how this barrier can be addressed (Strogatz et al., 2020; Syed et al., 2013). Transportation assistance for patients and their designated chaperones may be provided through volunteer driver programs, shuttle services, or other community programs. Several of the logistical barriers reported in this study were consistent with those described in the literature as barriers for surveillance in other cancer types, indicating that similar strategies for overcoming these barriers may be applied to PC surveillance (Franke et al., 2018; Nagelhout et al., 2017).

Patient recall of surveillance recommendations, another commonly reported barrier, also has high actionability. One possible explanation for the frequency of reported recall discrepancies in our study is the abundance of cancer surveillance and/or risk reduction recommendations provided to HRIs, particularly for individuals with PVs in genes that present an increased risk of cancer in multiple organs or organ systems. Addressing recall is especially important for HRIs receiving several recommendations at once and potentially from multiple healthcare professionals. Improved patient recall is associated with the use of recall‐promoting behaviors such as agenda setting, teach‐backs, and increased time spent on patients speaking during visits (Laws et al., 2018). A study of patients with Lynch syndrome identified that clear documentation of diagnosis in the medical record, easily accessible links to recommended surveillance in the EMR, and automated reminder prompts for both patients and healthcare professionals about upcoming or overdue surveillance all supported patient recall (Mittendorf et al., 2019). HRIs may not be aware of updated recommendations depending on the time elapsed since their last contact with the study site, and Mittendorf et al. (2019) additionally suggests the establishment of proactive, consistent outreach from genetics healthcare professionals to review care and surveillance recommendations against current guidelines. Utilizing this approach could support recall in HRIs as PC surveillance guidelines evolve.

There is continued difficulty in defining which individuals are or are not receiving appropriate surveillance given changing PC surveillance guidelines and an evolving evidence base. Surveillance practices differ between medical centers offering PC surveillance (unpublished data from the PRECEDE consortium) and between guidelines produced by different medical societies (Goggins et al., 2020; NCCN, 2025; Sawhney et al., 2022). HRIs in our study who reported different recommendations for surveillance may have received recommendations from a healthcare professional without expertise in PC surveillance or from an institution that follows a different set of surveillance guidelines. Further research is necessary to characterize differences between surveillance approaches and the reasons behind these differences; research in this area can support consistent and appropriate implementation of PC surveillance for HRIs. Consistent guidelines and surveillance practices are critical to ensure that HRIs receive appropriate surveillance at appropriate intervals, but these must be well‐informed by evidence demonstrating improved overall survival and balanced with the reduction of potential harms. HRIs undergoing PC surveillance should ideally be offered enrollment in a research study to allow longitudinal tracking of their surveillance outcomes to help researchers understand the limitations and challenges of PC surveillance in HRIs (Zogopoulos et al., 2024).

Cost, competing health concerns, nonmedical life events, moving, and COVID‐19 are not directly actionable by healthcare professionals working with HRIs, but consideration of these barriers highlights the limitations of current PC surveillance modalities. EUS cannot be completed at all hospitals capable of performing routine endoscopies, and moving or living farther away from a hospital equipped to perform EUS hinders surveillance completion; still, imaging study access may be addressed on a systems level by community hospitals that can implement PC surveillance programs (Raff et al., 2022). Annual EUS and/or MRI also present challenges beyond just logistics or cost, particularly for HRIs with chronic or competing health conditions. One study assessing barriers and facilitators to colonoscopy found that individuals with multiple chronic conditions (e.g., hypertension, hyperlipidemia, osteoarthritis, atherosclerotic heart disease, and diabetes mellitus) faced additional challenges when completing surveillance. Such challenges included preprocedure medication management and increased concerns for procedure‐related complications (Sultan et al., 2017). Individuals with heart disease, musculoskeletal disease, or lung disease may have shortness of breath while lying flat or have difficulty keeping still for MRIs. Aging increases the risk of chronic diseases, and the median age of our study population was 65 years. Given the inherent challenges of current PC surveillance modalities, it is critical to further investigate noninvasive screening tests, such as blood‐based tests, that may be easier to complete for HRIs and especially older adult HRIs.

This study had several limitations. While there were some differences in reported barriers between groups, there was not sufficient statistical power to determine whether significant differences existed in barriers between the cessation/deferral and noncommencement groups or based on factors within groups (e.g., differences in reported barriers for HRIs with PVs in PC risk genes versus HRIs with familial PC). Determining whether there are significant differences between HRIs who cease or defer PC surveillance compared with those who do not start surveillance is an area for future research. Our cohort was relatively small and was predominantly female and White; however, it is known that similar sex and racial disparities are present in populations throughout the PC surveillance field (Katona, 2023). Larger, multisite studies are necessary to generalize the results of this study to the broader population of HRIs undergoing PC surveillance, including HRIs from diverse backgrounds. Additionally, the cessation and deferral group participants in our study were identified from individuals enrolled in ongoing PC surveillance studies. HRIs not enrolled in research studies may have different reasons for deferring or ceasing PC surveillance than those who are enrolled in a research study. This cohort of participants was also enrolled in ongoing PC surveillance studies at varying time points between 2014 and 2021, a period in which guidelines for PC surveillance evolved significantly. Lastly, it was not always possible to compare participants' PC surveillance history with the specific recommendations from their healthcare professionals.

## 5 CONCLUSIONS

This study demonstrated that the most frequently cited reasons for cessation, deferral, or noncommencement of PC surveillance were logistical barriers, different provider recommendations, patient recall, or competing health concerns. Several of these barriers have actionable interventions. Other reasons for altered PC surveillance participation included imaging study concerns, contraindicating health issues, the COVID‐19 pandemic, moving away from the study site offering PC surveillance, nonmedical life events, and fear. While these other barriers are not directly actionable, research into noninvasive PC surveillance modalities may reduce their impact. Healthcare professionals, including genetic counselors, must be able to address patient concerns about PC surveillance. Even after genetic testing and results disclosure are performed, genetic counselors may still find themselves as a contact point for PC surveillance inquiries. Further research is necessary to understand reasons for cessation, deferral, or not starting PC surveillance in a broader population of HRIs, as well as the best ways to overcome PC surveillance challenges. Additional research about healthcare professionals' views of evolving PC surveillance guidelines may help support appropriate implementation of PC surveillance in HRIs across institutions. Together, our findings contribute to the limited literature focused on reasons for PC surveillance cessation, deferral, or noncommencement in HRIs.

## AUTHOR CONTRIBUTIONS

G.G.S., J.M.L., and B.W.K. conceptualized the study and designed the methodology. R.M. assisted with methodology design and formal analysis. G.G.S. conducted the investigation and formal analysis and authored the original draft. B.W.K., J.M.L., A.B., K.M., K.L.N., M.R., P.S., and S.M.D. provided resources for the project. D.C., S.K., and S.P. were responsible for project administration. G.G.S., J.M.L., and B.W.K. wrote the initial draft of the manuscript. All authors contributed to the reviewing and editing of the manuscript and approved the final version.

## CONFLICT OF INTEREST STATEMENT

B.W.K. receives clinical research funding from Janssen, Immunovia, Freenome, Guardant, Epigenomics, Universal Diagnostics, and Recursion and is on the advisory board for Immunovia. G.G.S., D.C., S.K., S.P., R.M., A.B., K.M., K.L.N., M.R., P.S., S.M.D., and J.M.L. declare that they have no conflict of interest.

## ETHICS STATEMENT

Human studies and informed consent: This study was approved by the Institutional Review Board of the University of Pennsylvania (protocol number 855599, approval date April 16, 2024). Verbal informed consent was obtained from participants to conduct interviews and to record their responses for inclusion in the study.

## Supporting information


Data S1:

Table S1:

## Data Availability

The data that support the findings of this study are not publicly available due to privacy concerns. Select parts of the data may be made available upon request to the corresponding author.

## References

[jgc470117-bib-0001] Blackford, A. L. , Canto, M. I. , Dbouk, M. , Hruban, R. H. , Katona, B. W. , Chak, A. , Brand, R. E. , Syngal, S. , Farrell, J. , Kastrinos, F. , Stoffel, E. M. , Rustgi, A. , Klein, A. P. , Kamel, I. , Fishman, E. K. , He, J. , Burkhart, R. , Shin, E. J. , Lennon, A. M. , & Goggins, M. (2024). Pancreatic cancer surveillance and survival of high‐risk individuals. JAMA Oncology, 10(8), 1087–1096. 10.1001/jamaoncol.2024.1930 38959011 PMC11223057

[jgc470117-bib-0002] Blackford, A. L. , Canto, M. I. , Klein, A. P. , Hruban, R. H. , & Goggins, M. (2020). Recent trends in the incidence and survival of stage 1A pancreatic cancer: A surveillance, epidemiology, and end results analysis. Journal of the National Cancer Institute, 112(11), 1162–1169. 10.1093/jnci/djaa004 31958122 PMC7669234

[jgc470117-bib-0003] Dbouk, M. , Katona, B. W. , Brand, R. E. , Chak, A. , Syngal, S. , Farrell, J. J. , Kastrinos, F. , Stoffel, E. M. , Blackford, A. L. , Rustgi, A. K. , Dudley, B. , Lee, L. S. , Chhoda, A. , Kwon, R. , Ginsberg, G. G. , Klein, A. P. , Kamel, I. , Hruban, R. H. , He, J. , … Goggins, M. (2022). The multicenter cancer of pancreas screening study: Impact on stage and survival. Journal of Clinical Oncology: Official Journal of the American Society of Clinical Oncology, 40(28), 3257–3266. 35704792 10.1200/JCO.22.00298 PMC9553376

[jgc470117-bib-0004] Dedoose . (2025). Dedoose Version 10.0.305, cloud application for managing, analyzing, and presenting qualitative and mixed method research data . SocioCultural Research Consultants, LLC. www.dedoose.com.

[jgc470117-bib-0005] Fendrich, V. , Langer, P. , & Bartsch, D. K. (2014). Familial pancreatic cancer–status quo. International Journal of Colorectal Disease, 29(2), 139–145. 10.1007/s00384-013-1760-3 23948969

[jgc470117-bib-0006] Franke, F. S. , Matthäi, E. , Slater, E. P. , Schicker, C. , Kruse, J. , & Bartsch, D. K. (2018). German National Case Collection for familial pancreatic cancer (FaPaCa) – Acceptance and psychological aspects of a pancreatic cancer screening program 17 psychology and cognitive sciences 1701 psychology. Hereditary Cancer in Clinical Practice, 16(1), 17. 10.1186/s13053-018-0100-6 30519369 PMC6267785

[jgc470117-bib-0007] Goggins, M. , Overbeek, K. A. , Brand, R. , Syngal, S. , del Chiaro, M. , Bartsch, D. K. , Bassi, C. , Carrato, A. , Farrell, J. , Fishman, E. K. , Fockens, P. , Gress, T. M. , van Hooft, J. E. , Hruban, R. H. , Kastrinos, F. , Klein, A. , Lennon, A. M. , Lucas, A. , Park, W. , … Bruno, M. (2020). Management of patients with increased risk for familial pancreatic cancer: Updated recommendations from the international cancer of the pancreas screening (CAPS) consortium. Gut, 69(1), 7–17. 10.1136/gutjnl-2019-319352 31672839 PMC7295005

[jgc470117-bib-0008] Hsieh, H. F. , & Shannon, S. E. (2005). Three approaches to qualitative content analysis. Qualitative Health Research, 15(9), 1277–1288. 16204405 10.1177/1049732305276687

[jgc470117-bib-0009] Katona, B. W. , Klute, K. , Brand, R. E. , Everett, J. N. , Farrell, J. J. , Hawthorne, K. , Kaul, V. , Kupfer, S. S. , Paiella, S. , Simeone, D. M. , Sussman, D. A. , Zogopoulos, G. , Lucas, A. L. , Kastrinos, F. , & PRECEDE Consortium . (2023). Racial, ethnic, and sex‐based disparities among high‐risk individuals undergoing pancreatic cancer surveillance. Cancer Prevention Research (Philadelphia, Pa.), 16(6), 343–352. 10.1158/1940-6207.CAPR-22-0529 37259800 PMC12380199

[jgc470117-bib-0010] Klatte, D. C. F. , Boekestijn, B. , Onnekink, A. M. , Dekker, F. W. , van der Geest, L. G. , Wasser, M. N. J. M. , Feshtali, S. , Mieog, J. S. D. , Luelmo, S. A. C. , Morreau, H. , Potjer, T. P. , Inderson, A. , Boonstra, J. J. , Vasen, H. F. A. , van Hooft, J. E. , Bonsing, B. A. , van Leerdam, M. E. , & Dutch Pancreatic Cancer Group . (2023). Surveillance for pancreatic cancer in high‐risk individuals leads to improved outcomes: A propensity score‐matched analysis. Gastroenterology, 164(7), 1223–1231.e4. 10.1053/j.gastro.2023.02.032 36889551

[jgc470117-bib-0011] Laws, M. B. , Lee, Y. , Taubin, T. , Rogers, W. H. , & Wilson, I. B. (2018). Factors associated with patient recall of key information in ambulatory specialty care visits: Results of an innovative methodology. PLoS One, 13(2), e0191940. 10.1371/journal.pone.0191940 29389994 PMC5794108

[jgc470117-bib-0012] Lynch, F. , Gillam, L. , & Vears, D. F. (2024). Alleviating the confusion around content analysis: A comment in response to Wainstein, Elliott & Austin 2023. Journal of Genetic Counseling, 33(5), 1126–1129. 10.1002/jgc4.1829 37877543

[jgc470117-bib-0013] Mittendorf, K. F. , Hunter, J. E. , Schneider, J. L. , Shuster, E. , Rope, A. F. , Zepp, J. , Gilmore, M. J. , Muessig, K. R. , Davis, J. V. , Kauffman, T. L. , Bergen, K. M. , Wiesner, G. L. , Acheson, L. S. , Peterson, S. K. , Syngal, S. , Reiss, J. A. , & Goddard, K. A. B. (2019). Recommended care and care adherence following a diagnosis of Lynch syndrome: A mixed‐methods study. Hereditary Cancer in Clinical Practice, 17, 31. 10.1186/s13053-019-0130-8 31890059 PMC6915941

[jgc470117-bib-0014] Mooney, R. , Wu, Y. P. , Kehoe, K. , Volkmar, M. , Kohlmann, W. , Koptiuch, C. , & Kaphingst, K. A. (2023). Experiences of patients and family members with follow‐up care, information needs and provider support after identification of Lynch syndrome. Hereditary Cancer in Clinical Practice, 21(1), 28. 10.1186/s13053-023-00273-1 38115072 PMC10731879

[jgc470117-bib-0015] Nagelhout, E. , Comarell, K. , Samadder, N. J. , & Wu, Y. P. (2017). Barriers to colorectal cancer screening in a racially diverse population served by a safety‐net clinic. Journal of Community Health, 42(4), 791–796. 10.1007/s10900-017-0319-6 28168395 PMC5517041

[jgc470117-bib-0016] National Comprehensive Cancer Network . (2025). National Comprehensive Cancer Network Guidelines – Genetic/familial high‐risk assessment: Breast, ovarian, pancreatic, and prostate, version 3.2025 . https://www.nccn.org/guidelines/guidelines‐detail?category=2&id=1545 10.6004/jnccn.2026.0007 41671423

[jgc470117-bib-0017] Owens, D. K. , Davidson, K. W. , Krist, A. H. , Barry, M. J. , Cabana, M. , Caughey, A. B. , Curry, S. J. , Doubeni, C. A. , Epling, J. W. , Kubik, M. , Landefeld, C. S. , Mangione, C. M. , Pbert, L. , Silverstein, M. , Simon, M. A. , Tseng, C. W. , & Wong, J. B. (2019). Screening for pancreatic cancer: US preventive services task force reaffirmation recommendation Statement. JAMA, 322(5), 438–444. 10.1001/jama.2019.10232 31386141

[jgc470117-bib-0018] Raff, J. P. , Cook, B. , Jafri, F. N. , Boxer, N. , Maldonado, J. , Hopkins, U. , Roayaie, S. , & Noyer, C. (2022). Successful pancreatic cancer screening among individuals at elevated risk using endoscopic ultrasound and magnetic resonance imaging: A community hospital experience. Pancreas, 51(10), 1345–1351. 10.1097/MPA.0000000000002182 37099777

[jgc470117-bib-0019] Rahib, L. , Wehner, M. R. , Matrisian, L. M. , & Nead, K. T. (2021). Estimated projection of US cancer incidence and death to 2040. JAMA Network Open, 4(4), e214708. 10.1001/jamanetworkopen.2021.4708 33825840 PMC8027914

[jgc470117-bib-0021] Sawhney, M. S. , Calderwood, A. H. , Thosani, N. C. , Rebbeck, T. R. , Wani, S. , Canto, M. I. , Fishman, D. S. , Golan, T. , Hidalgo, M. , Kwon, R. S. , Riegert‐Johnson, D. L. , Sahani, D. v. , Stoffel, E. M. , Vollmer, C. M. , & Qumseya, B. J. (2022). ASGE guideline on screening for pancreatic cancer in individuals with genetic susceptibility: Summary and recommendations. Gastrointestinal Endoscopy, 95(5), 817–826. 10.1016/j.gie.2021.12.001 35183358

[jgc470117-bib-0022] Seppälä, T. T. , Burkhart, R. A. , & Katona, B. W. (2023). Hereditary colorectal, gastric, and pancreatic cancer: Comprehensive review. BJS Open, 7(3), zrad023. 10.1093/bjsopen/zrad023 37165697 PMC10172688

[jgc470117-bib-0030] Siegel, R. L. , Giaquinto, A. N. , & Jemal, A. , (2024). Cancer statistics, 2024. CA: A Cancer Journal for Clinicians, 74(1), 12–49. 10.3322/caac.21820 38230766

[jgc470117-bib-0029] Siegel, R. L. , Kratzer, T. B. , Giaquinto, A. N. , Sung, H. , & Jemal, A . (2025). Cancer statistics, 2025. CA: A Cancer Journal for Clinicians, 75(1), 10–45. 10.3322/caac.21871 39817679 PMC11745215

[jgc470117-bib-0023] Strogatz, D. , Mielenz, T. J. , Johnson, A. K. , Baker, I. R. , Robinson, M. , Mebust, S. P. , Andrews, H. F. , Betz, M. E. , Eby, D. W. , Johnson, R. M. , Jones, V. C. , Leu, C. S. , Molnar, L. J. , Rebok, G. W. , & Li, G. (2020). Importance of driving and potential impact of driving cessation for rural and urban older adults. The Journal of Rural Health, 36(1), 88–93. 10.1111/jrh.12369 31022317

[jgc470117-bib-0024] Sultan, S. , Partin, M. R. , Shah, P. , LeLaurin, J. , Freytes, I. M. , Nightingale, C. L. , Fesperman, S. F. , Curbow, B. A. , & Beyth, R. J. (2017). Barriers and facilitators associated with colonoscopy completion in individuals with multiple chronic conditions: A qualitative study. Patient Preference and Adherence, 11, 985–994. 10.2147/PPA.S127862 28579761 PMC5449171

[jgc470117-bib-0025] Syed, S. T. , Gerber, B. S. , & Sharp, L. K. (2013). Traveling towards disease: Transportation barriers to health care access. Journal of Community Health, 38(5), 976–993. 10.1007/s10900-013-9681-1 23543372 PMC4265215

[jgc470117-bib-0026] Syngal, S. , Brand, R. E. , Church, J. M. , Giardiello, F. M. , Hampel, H. L. , & Burt, R. W. (2015). ACG clinical guideline: Genetic testing and management of hereditary gastrointestinal cancer syndromes. American Journal of Gastroenterology, 110(2), 223–262. 10.1038/ajg.2014.435 25645574 PMC4695986

[jgc470117-bib-0027] Wood, L. D. , Yurgelun, M. B. , & Goggins, M. G. (2019). Genetics of familial and sporadic pancreatic cancer. Gastroenterology, 156(7), 2041–2055. 10.1053/j.gastro.2018.12.039 30660730

[jgc470117-bib-0028] Zogopoulos, G. , Haimi, I. , Sanoba, S. A. , Everett, J. N. , Wang, Y. , Katona, B. W. , Farrell, J. J. , Grossberg, A. J. , Paiella, S. , Klute, K. A. , Bi, Y. , Wallace, M. B. , Kwon, R. S. , Stoffel, E. M. , Wadlow, R. C. , Sussman, D. A. , Merchant, N. B. , Permuth, J. B. , Golan, T. , … Simeone, D. M. (2024). The pancreatic cancer early detection (PRECEDE) study is a global effort to drive early detection: Baseline imaging findings in high‐risk individuals. Journal of the National Comprehensive Cancer Network, 22(3), 158–166. 10.6004/jnccn.2023.7097 38626807 PMC12344727

