# Supplementary material for: Assessment of barriers to pancreatic cancer surveillance in high‐risk individuals
Source: J Genet Couns. 2025 Oct 9;34(5):e70117. doi: 10.1002/jgc4.70117 (PMC12511838; doi:10.1002/jgc4.70117)
Supplement: Supplementary file 1 — Data S1: [file JGC4-34-0-s001.docx]

**Interview Script**

For patients who have not had imaging in >=2 years:

In review of our records, we noticed that it has been more than 2 years since your last pancreatic cancer screening exam. The date of your last exam we have on file is [insert date]. Was this the last time you had pancreatic cancer screening?

If No 🡪 Where else did you have pancreatic cancer screening done? What were the factors that led you to seek care at [location]?

If Yes 🡪 What are the reasons you would say you have not had any recent pancreatic cancer screening?

1. Have there been any major changes in your medical history since your last pancreatic cancer screening?
   1. If Yes: Have these changes impacted your ability to undergo pancreatic cancer screening?
2. Have there been any other major changes in your life since your last screening?
   1. If Yes: Have these changes impacted your ability to undergo pancreatic cancer screening?
3. Is there anything else that has prevented or discouraged you from scheduling pancreatic cancer screening since your last visit?
   1. Prompt 🡪 This could be anything: emotional, financial, or physical.
   2. If Yes: Have these changes impacted your ability to undergo pancreatic cancer screening?

For patients who had a gap in imaging of at least 2 years:

In review of our records we saw that you had a period of at least 2 years where you did not receive any pancreatic cancer screening. [Prompt with specific years if helpful for recall]. Did you receive cancer screening at a different institution during this time?

If Yes 🡪 What were the factors that led you to seek care at [location]?
If No 🡪 Do you recall the reasons that you did not undergo pancreatic cancer screening during that period? What were those reasons?

1. Were there any major changes in your medical history during [time period]?
   1. If Yes: Did those changes impact your ability to undergo pancreatic cancer screening?
2. Were there any other major changes in your life during that time period?
   1. If Yes: Did those changes impact your ability to undergo pancreatic cancer screening?
3. Was there anything else that prevented or discouraged you from scheduling or attending a screening procedure during that time?
   1. Prompt 🡪 This could be anything: emotional, financial, or physical.
   2. If Yes: Did those changes impact your ability to undergo pancreatic cancer screening?

For patients who were recommended to begin surveillance but have not had imaging:

In review of our records we noticed that pancreatic cancer screening had been ordered for you by one of your providers [give name of the provider who ordered the study if needed]. However, in our records we did not see that you had ever undergone a pancreatic cancer screening test such as an MRI or endoscopic ultrasound.

1. Do you recall your doctor making a recommendation to receive one of these pancreatic screening tests?
2. Have you ever received a pancreatic cancer screening procedure, like MRI or EUS?
   1. If Yes 🡪 What were the factors that led you to seek care at [location]?
   2. If No 🡪 What are the reasons that you would say you did not receive pancreatic cancer screening?
3. Were there any major changes in your life that happened shortly before or after your doctor recommended starting screening?
   1. If Yes: Did those changes impact your ability to undergo pancreatic cancer screening?
4. Is there anything else that prevented or discouraged you from scheduling pancreatic cancer screening after your doctor’s initial recommendation?
   1. Prompt 🡪 This could be anything: emotional, financial, or physical.
   2. If Yes: Have those changes impacted your ability to undergo pancreatic cancer screening?

Final questions for all groups:

Are there any other issues you have faced while accessing or scheduling pancreatic cancer screening that you would like to report? [If expressed interest] Is there anything we can do to help with scheduling or continuing your pancreatic cancer screening?

**Interview Closure**

Thank you so much for your time. Your responses will help us improve patient access to pancreatic cancer screening.
