# Supplementary material for: Assessment of barriers to pancreatic cancer surveillance in high‐risk individuals
Source: J Genet Couns. 2025 Oct 9;34(5):e70117. doi: 10.1002/jgc4.70117 (PMC12511838; doi:10.1002/jgc4.70117)
Supplement: Supplementary file 2 — Table S1: [file JGC4-34-0-s002.docx]

|  | **Total** | **Participated in interview** | **Did not participate** |
| --- | --- | --- | --- |
| ***BRCA2*** | **32** | **15** | **17** |
| (+) Positive Family History | 17 | 8 | 9 |
| (-) Negative/Unknown Family History | 15 | 7 | 8 |
| ***BRCA1*** | **12** | **8** | **4** |
| (+) Positive Family History | 3 | 2 | 1 |
| (-) Negative/Unknown Family History | 9 | 6 | 3 |
| ***ATM*** | **8** | **7** | **1** |
| (+) Positive Family History | 3 | 2 | 1 |
| (-) Negative/Unknown Family History | 5 | 5 | 0 |
| **Lynch syndrome, excluding *PMS2* (*MLH1, MSH2/EPCAM,* or *MSH6)*** | **6** | **3** | **3** |
| (+) Positive Family History | 4 | 2 | 2 |
| (-) Negative/Unknown Family History | 2 | 1 | 1 |
| ***CDKN2A*** | **5** | **1** | **4** |
| (-) Negative/Unknown Family History | 5 | 1 | 4 |
| ***PALB2*** | **4** | **3** | **1** |
| (+) Positive Family History | 2 | 1 | 1 |
| (-) Negative/Unknown Family History | 2 | 2 | 0 |
| ***STK11*** | **1** | **1** | **0** |
| (+) Positive Family History | 1 | 1 | 0 |

**Supplemental Table 1: Family history of PC in participants with PVs in a PC risk gene.** Of the total 68 potential participants who had a PV in a PC risk gene, 30 had a positive family history and 38 had a negative or unknown family history of PC. Of those who participated, 16 had a positive family history and 22 had a negative or unknown family history. Of those who did not participate, 14 had a positive family history and 16 had a negative or unknown family history.
